# *Toxoplasma* TgAtg8-TgAtg3 Interaction Primarily Contributes to Apicoplast Inheritance and Parasite Growth in Tachyzoite

**DOI:** 10.1128/spectrum.01495-21

**Published:** 2022-02-23

**Authors:** Linyan Cheng, Yuan Tian, Yan Wang, Tingting Wang, Yinning Yao, Helin Yu, Xiaozi Zheng, Mimi Wu, Wei Zhao, Qianqian Hua, Xin Hu, Feng Tan

**Affiliations:** a School of the First Clinical Medical Sciences, Wenzhou Medical University, Wenzhou, Zhejiang, China; b Department of Parasitology, School of Basic Medical Sciences, Wenzhou Medical University, Wenzhou, Zhejiang, China; c Teaching Center of Morphological Experiment, School of Basic Medical Sciences, Wenzhou Medical University, Wenzhou, Zhejiang, China; d Clinical Laboratory, Dongyang People's Hospital, Jinhua, Zhejiang, China; e School of Medical Laboratory Science and School of Life Science, Wenzhou Medical University, Wenzhou, Zhejiang, China; Hebrew University of Jerusalem

**Keywords:** *Toxoplasma*, autophagy, Atg8, Atg3, protein-protein interaction, Atg8-family interacting motif, apicoplast

## Abstract

The apicoplast, which harbors key pathways involved in biosynthesis of vital metabolites, is a unique and essential nonphotosynthetic plastid organelle in apicomplexan parasites. Intriguingly, autophagy-related protein 8 (Atg8), a highly conserved eukaryotic protein, can localize to the outermost membrane of the apicoplast and modulate its inheritance in both *Toxoplasma* and *Plasmodium* parasites. The Atg8-Atg3 interaction plays a key role in Atg8 lipidation and localization, and our previously work in *Toxoplasma* has suggested that the core Atg8-family interacting motif (AIM) in TgAtg3, ^239^FADI^242^, and the R27 residue of TgAtg8 contribute to TgAtg8-TgAtg3 interaction *in vitro*. However, little is known about the function of this interaction or its importance in tachyzoite growth in *Toxoplasma gondii*. Here, we generated two complemented cell lines, TgAtg3^F239A/I242A^ and TgAtg8^R27E^, based on the TgAtg3 and TgAtg8 conditional knockdown cell lines, respectively. We found that both mutant complemented cell lines were severely affected in terms of tachyzoite growth and displayed delayed death upon conditional knockdown of endogenous TgAtg3 or TgAtg8. Intriguingly, both complemented lines appeared to be defective in TgAtg8 lipidation and apicoplast inheritance. Moreover, we showed that the interaction of TgAtg8 and TgAtg3 is critical for TgAtg8 apicoplast localization. In addition, we found that the TgAtg3^F239A/I242A^ complemented line exhibits an integral mitochondrial network upon ablation of endogenous TgAtg3, which is distinct from TgAtg3-depleted parasites with a fragmented mitochondrial network. Taken together, this work solidifies the contribution of the TgAtg8-TgAtg3 interaction to apicoplast inheritance and the growth of *T. gondii* tachyzoites.

**IMPORTANCE** *Toxoplasma gondii*is a widespread intracellular parasite infecting a variety of warm-blooded animals, including humans. Current frontline treatment of toxoplasmosis suffers many drawbacks, including toxicity, drug resistance, and failure to eradicate tissue cysts, underscoring the need to identify novel drug targets for suppression or treatment of toxoplasmosis. TgAtg8 is thought to serve multiple functions in lipidation and is considered essential to the growth and development of both tachyzoites and bradyzoites. Here, we show that *Toxoplasma gondii* has adapted a conserved Atg8-Atg3 interaction, required for canonical autophagy in other eukaryotes, to function specifically in apicoplast inheritance. Our finding not only highlights the importance of TgAtg8-TgAtg3 interaction in tachyzoite growth but also suggests that this interaction is a promising drug target for the therapy of toxoplasmosis.

## INTRODUCTION

Toxoplasma gondii belongs to the phylum Apicomplexa, which includes several important human parasites, such as *Plasmodium* spp., the causative agent of malaria (1). Toxoplasma gondii is an obligate intracellular parasite of warm-blooded animals that can cause zoonotic toxoplasmosis (2). Early infection during pregnancy can lead to birth defects, miscarriage, and neurological pathologies since the parasite can infect the developing fetus via the placenta. Also, *Toxoplasma* infection can be lethal for immunocompromised patients (3–5). Deficiencies such as limited therapeutic efficacy, serious side effects, and increasing drug resistance (6–8) have been noted in current conventional anti-*Toxoplasma* drugs, highlighting the importance of continued identification of effective drug targets.

Macroautophagy (here referred to as autophagy) is an intracellular catabolic process in eukaryotic cells. One of the main functions of autophagy is the maintenance of basal cellular homeostasis under normal growth conditions. On the other hand, autophagy is crucial for stress management in almost all eukaryotes under diverse stress conditions. Hypoxia, reactive oxygen species, or deficiency of nutrients such as amino acids or glucose are known to be important drivers of autophagy, which rallies cellular energy and nutrient stores to sustain normal cell functions (9–11). During the autophagic process, cytosolic components such as misfolded proteins or damaged organelles are sequestered within a double-membrane vesicle called an autophagosome and eventually delivered into lysosome for degradation (11, 12). Autophagy-related (Atg) 8 protein is a key modulator of autophagosomal formation by facilitating membrane elongation and fusion via lipid conjugation to phosphatidylethanolamine (PE) (13). The relocalization of Atg8 from dispersing in the cytoplasm to forming autophagosomal vesicular structures, and the characteristic protein band-shift between unlipidated (Atg8) and lipidated (Atg8-PE) forms, are widely used to define and assay autophagy-related processes (13).

It has previously been shown that autophagosome-like structures can be observed in response to nutrient starvation and endoplasmic reticulum stress in tachyzoites (14, 15) and bradyzoites (16, 17) of *Toxoplasma*. However, besides its cytosolic and autophagosomal localizations, the Toxoplasma gondii Atg8 (TgAtg8) homologue also localizes to the outermost membrane of the apicoplast, a nonphotosynthetic plastid-like organelle shared by most members of Apicomplexa (18). The apicoplast is surrounded by four membranes and is essential for parasite survival due to its important functions in several key metabolic pathways (19). During each parasite division cycle, the parental apicoplast needs to be segregated into each daughter cell for proper inheritance across generations. Defects in apicoplast segregation will cause the loss of apicoplast in daughter cells and subsequently result in the “delayed death” phenotype (20, 21). As reported previously, TgAtg8 plays a vital role in maintaining apicoplast inheritance by tethering the apicoplast to centrosomes, either directly or indirectly, to guarantee proper segregation of this organelle during parasite division (18).

The lipidation of Atg8 is carried out via the E1-like Atg7 and the E2-like Atg3, with the E3-like Atg5-Atg12-Atg16 complex the stimulating transfer of Atg8 from Atg3 to PE. In addition, Atg4 is responsible for both cleaving the C terminus of Atg8 to expose a glycine residue for lipidation and for liberating Atg8 from PE during autophagosome maturation (22). Interestingly, TgAtg8 (18) and its lipid conjugation machinery, such as TgAtg4 (23), TgAtg3 (23), and TgAtg7 (our unpublished data), have been shown to be important for apicoplast inheritance, implying that the autophagy-related machinery in *Toxoplasma* has been repurposed for a noncanonical function associated with this organelle.

Atg8-Atg3 interaction is critical in the regulation of Atg8 lipidation, in which Atg3 noncovalently interacts with Atg8 through an Atg8-family interacting motif (AIM) in Atg3 and two hydrophobic pockets (the W- and L-sites) in Atg8 (24). Several studies in Plasmodium falciparum have suggested that the Atg8-Atg3 interaction may be an attractive new drug target in apicomplexan parasites (25–27). Using a series of biochemistry assays *in vitro*, we have previously verified the importance of the TgAtg8-TgAtg3 interaction in TgAtg8 lipidation and demonstrated that the core AIM of TgAtg3, ^239^FADI^242^, which is distinct from *Plasmodium*, plays an important role in mediating this interaction (28); however, the function of the TgAtg8-TgAtg3 interaction in the *Toxoplasma* life cycle remains poorly understood. Based on our previous findings, we separately generated TgAtg3^F293A/I242A^ and TgAtg8^R27E^ mutants in this study to provide the first direct evidence that the TgAtg8-TgAtg3 interaction is essential to *in vivo* TgAtg8 lipidation and apicoplast inheritance, and that disruption of this interaction leads to the delayed death phenotype.

## RESULTS

### Genetic knockdown of endogenous TgAtg3 and complementation of TgAtg3^AIM^ mutant.

**FIG 1:**
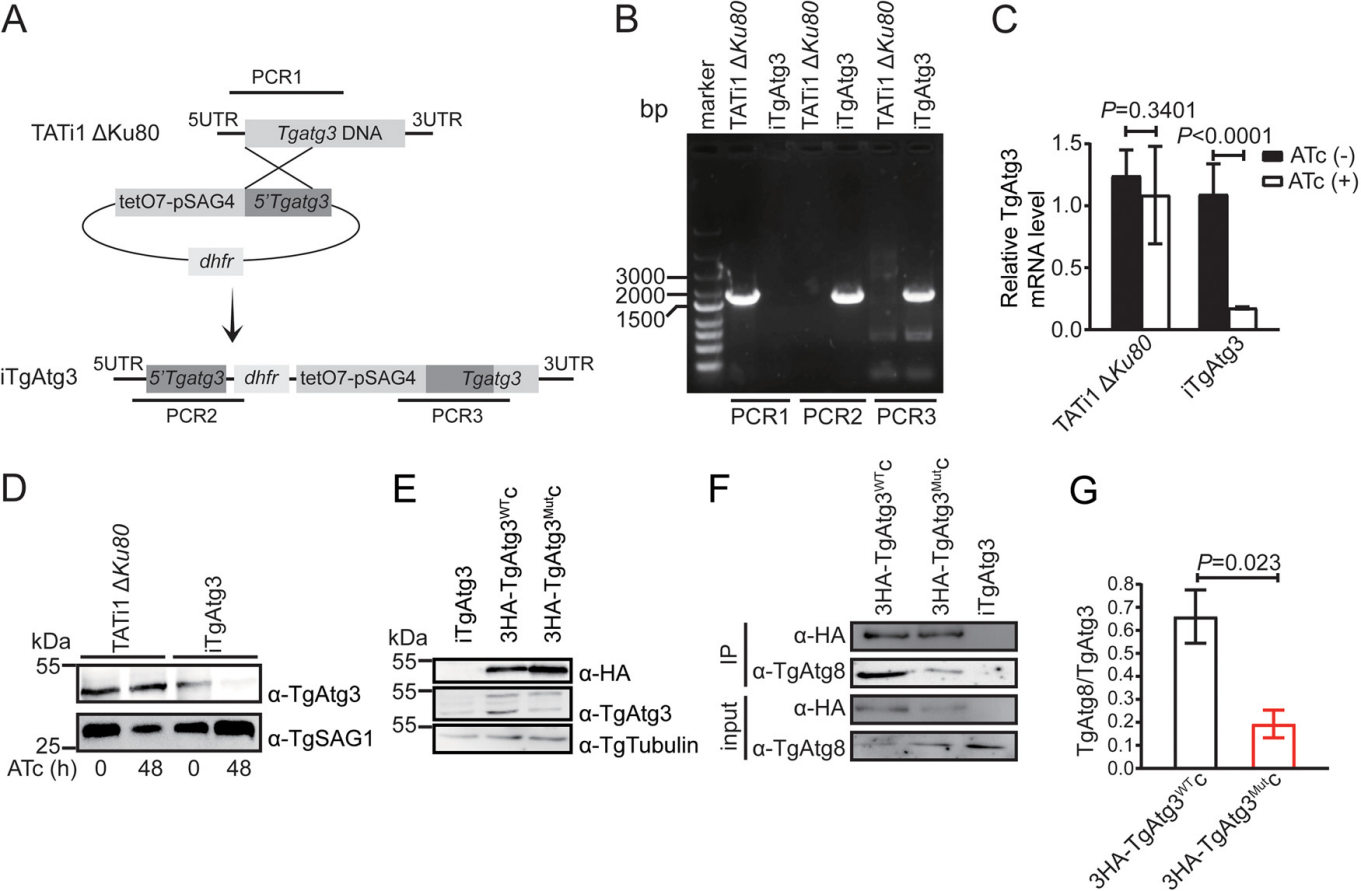
Generation of iTgAtg3 and complemented line. (A) Schematic representation of the linear knockdown construct and generation of the inducible TgAtg3 knockdown line by replacement of the endogenous promoter with an ATc-regulated promoter in a parental TATi1 Δ*Ku80* background. TATi1, transactivator; tetO7, *tet* operator; *dhfr*, DHFR selection marker; pSAG4, SAG4 minimal promoter. (B) Clone derived from a stable pyrimethamine-resistant population was detected for endogenous and recombined loci by amplifying the sequences shown in panel A using diagnostic PCR. TATi1 Δ*Ku80* line is used as control. Three independent experiments were performed. (C) RT-qPCR analyses of *TgAtg3* transcriptional level in parental and iTgAtg3 lines, preceded or not by 48 h of induction with ATc to regulate expression. Data are means from *n* = 3 independent experiments ± SEM. Statistical analysis was done using Student's *t* test. (D) WB analyses of TgAtg3 expression in protein extracts from parental and iTgAtg3 lines after 0 and 48 h of ATc treatment, using anti-TgAtg3 antibody. The product of the regulated *TgAtg3* gene is detectable in the absence of ATc and shows severe reduction after 48 h. Tubulin serves as loading control. (E) WB analyses of TgAtg3 expression in protein extracts from iTgAtg3 and two complemented lines (3HA-TgAtg3^WT^c and 3HA-TgAtg3^Mut^c) without ATc treatment. Both anti-TgAtg3 and anti-HA antibodies show 3HA-fused TgAtg3 protein in the two complemented lines, and anti-TgAtg3 antibody also reveals endogenous TgAtg3 protein in all lines. Anti-tubulin antibody serves as loading control. Results of three independent experiments are shown in panels D and E. (F) *In vivo* immunoprecipitation using HA antibodies. Protein extracts from either iTgAtg3, 3HA-TgAtg3^WT^c, or 3HA-TgAtg3^Mut^c cell lines, without ATc treatment, were immunoprecipitated separately with anti-HA beads. Inputs and IP proteins were analyzed by WB using the indicated antibodies. Two independent experiments with a combination of *in vivo* immunoprecipitation and WB were performed. Image represents one of two independent experiments. (G) Binding was quantified with ImageJ as the ratio of bound TgAtg8 to TgAtg3. Data are means from *n* = 2 independent experiments ± SEM. Statistical analysis was done using Student's *t* test.

Our previous work focused on identifying the core AIM in TgAtg3 through biochemical analyses *in vitro* and determined that the ^239^FADI^242^ of TgAtg3 is responsible for mediating TgAtg8-TgAtg3 interaction (28). To evaluate whether the TgAtg8-TgAtg3 interaction is involved in tachyzoite growth and apicoplast inheritance, we first generated a conditionally inducible knockdown cell line of TgAtg3 (iTgAtg3) using a transactivator (TATi)-based, tetracycline (Tet)-regulated system (29, 30). The endogenous promoter of *TgAtg3* was replaced successfully with a T7S4 promoter, a hybrid of the promoter of *Toxoplasma sag4* gene and the anhydrotetracycline (ATc)-responsive promoter (Fig. 1A), using diagnostic PCR (Fig. 1B). Real-time quantitative PCR (RT-qPCR) analyses showed that *TgAtg3* transcripts in the iTgAtg3 line were significantly downregulated 48 h after ATc treatment (non-treatment versus treatment: 0.175 ± 0.002 versus 1.087 ± 0.091) (Fig. 1C). Moreover, the TgAtg3 protein, expressed at the expected molecular mass of ∼45 kDa, in the resulting knockdown line was below the limit of detection 48 h after treatment with ATc (Fig. 1D).

The iTgAtg3 line was then complemented with either N-terminal, triple-hemagglutinin (3HA)-tagged, wild-type (WT) gene (3HA-TgAtg3^WT^c) or a mutant allele (3HA-TgAtg3^Mut^c) which harbored F293A/I242A double-point mutations identified to disrupt TgAtg8-TgAtg3 interaction (28). These constructs were integrated into the uracil phosphoribosyltransferase (UPRT) locus by double-homologous recombination using CRISPR/Cas9, and incorporation into the genome was confirmed by PCR detection and sequencing (Fig. S1A and B in the supplemental material). Moreover, using the anti-HA antibody, the complemented cell lines were validated by the detection of 3HA-fused TgAtg3 protein, expressed at the expected molecular mass of ∼50 kDa. We also confirmed the native TgAtg3 (∼45 kDa) and 3HA-TgAtg3 protein (∼50 kDa) in both complemented cell lines using a specific anti-TgAtg3 antibody (Fig. 1E).

Our previous study showed that the mutation of TgAtg3 AIM significantly reduces the ability of TgAtg3 to bind to TgAtg8 *in vitro* (28). In the present study, we evaluated further the impact of TgAtg3 AIM on TgAtg8-TgAtg3 interaction using both 3HA-TgAtg3^WT^c and 3HA-TgAtg3^Mut^c strains via *in vivo* immunoprecipitation. Indeed, we found that the mutation of AIM in TgAtg3 impairs TgAtg8-TgAtg3 interaction *in vivo*, as a detectable reduction in the 14 kDa product corresponding to TgAtg8 was shown in 3HA-TgAtg3^Mut^c strains (0.19 ± 0.06) compared to 3HA-TgAtg3^WT^c (0.66 ± 0.12) (Fig. 1F and G).

### TgAtg3 AIM mutants show impairment of TgAtg8 lipidation.

**FIG 2:**
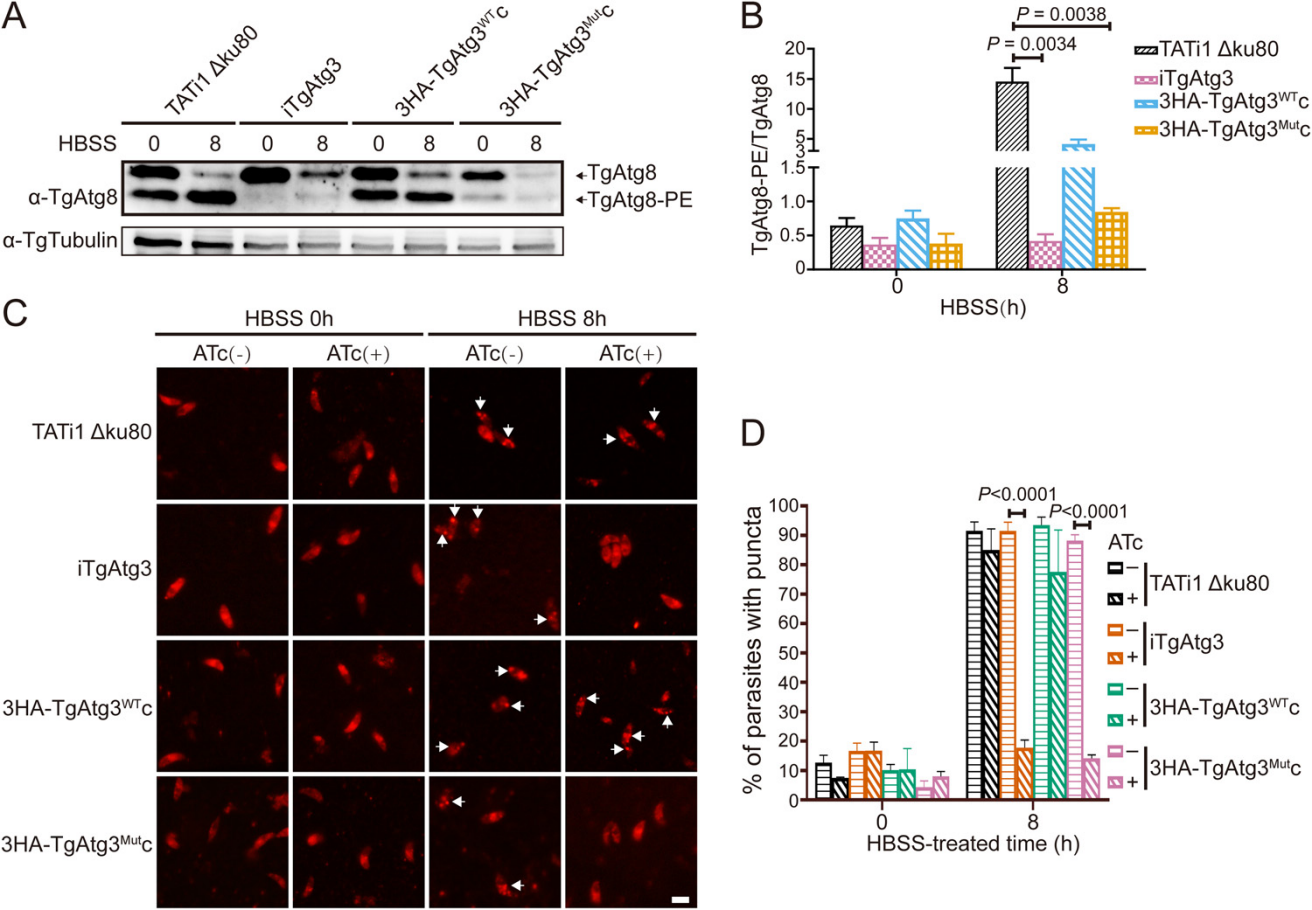
TgAtg3 AIM mutant reduces TgAtg8 lipidation. (A) Urea-SDS-PAGE and WB analyses of unmodified or PE-conjugated forms of TgAtg8 in TATi1 Δ*Ku80*, iTgAtg3, 3HA-TgAtg3^WT^c, and 3HA-TgAtg3^Mut^c lines, preincubated with ATc for 48 h and either unstarved or put in starvation medium for 8 h. Anti-tubulin serves as loading control. (B) Band densitometry quantification of the ratio of TgAtg8-PE to TgAtg8. Data are means from *n *=* *3 independent experiments ± SEM. Statistical analysis was done using Student's *t* test. (C) TATi1 Δ*Ku80*, iTgAtg3, 3HA-TgAtg3^WT^c, and 3HA-TgAtg3^Mut^c lines were preincubated for 48 h in the absence or presence of ATc, and then their extracellular tachyzoites were put to starve in HBSS medium for 8 h, respectively. Subsequently, IFA assay was performed to detect TgAtg8 puncta in all lines using anti-TgAtg8 antibody. Puncta are labeled with white arrowheads. Scale bar = 5 μm. (D) Proportions of parasites displaying punctate or cytosolic TgAtg8 signals were assessed. Data are means from *n *=* *3 independent experiments ± SEM. Statistical analysis was done using one-way ANOVA.

Due to the importance of the TgAtg8-TgAtg3 interaction in TgAtg8 lipidation, we sought to evaluate the effect of the TgAtg3^F239A/I242A^ mutant version on TgAtg8-PE formation using Western blotting (WB) and an immunofluorescence assay (IFA) with anti-TgAtg8 antibody. All cell lines were treated with ATc for 48 h, and then the fresh extracellular parasites were kept in Hanks’ Balanced Salt Solution (HBSS) for 8h to induce autophagy. WB analyses showed that while the lipidated form of TgAtg8 increased following starvation induction in the parental (14.55 ± 2.27) and 3HA-TgAtg3^WT^c (4.15 ± 0.75) lines, TgAtg8 lipidation was significantly impaired upon TgAtg3 depletion in the iTgAtg3 (0.42 ± 0.10) and 3HA-TgAtg3^Mut^c (0.85 ± 0.05) lines, as the intensity of the band corresponding to TgAtg8-PE was markedly reduced (TgAtg8-PE/TgAtg8 < 1) (Fig. 2A and B). In addition, we noticed that a detectable TgAtg8-PE band, strongly existing in the parental and 3HA-TgAtg3^WT^c lines, was very weak in the iTgAtg3 and 3HA-TgAtg3^Mut^c lines at 0 h. Previous reports have mentioned that native TgAtg8 appears to already exist in significant proportions as a lipidated form under basal conditions (14, 23). Moreover, TgAtg8 has been shown to be localized to the outer membrane of the apicoplast through an association with PE in T. gondii tachyzoites (18). We therefore assumed that disruption of the TgAtg8-TgAtg3 interaction probably hindered the apicoplast localization of TgAtg8.

Nonetheless, as shown in Fig. 2A, we noticed that depletion of endogenous TgAtg3 in either the iTgAtg3 or the 3HA-TgAtg3^Mut^c lines seemed to result in a significant reduction in TgAtg8 protein levels. A previous study indicated that TgAtg8 at lower levels can still accomplish its cellular function (18); however, to rule out the potential impact of reduced TgAtg8 protein levels on its function, all cell lines were preincubated for 48 h in the absence or presence of ATc, and then their extracellular tachyzoites were put to starve in HBSS medium for 8 h, respectively. Subsequently, IFA was performed to detect TgAtg8 puncta using anti-TgAtg8 antibody. We found that in the absence of ATc, nutrient starvation caused a significant amount of TgAtg8 punctate localization in all lines, indicating that TgAtg8 can accomplish its lipidation (Fig. 2C and D). Furthermore, the IFA results showed that the number of parasites containing TgAtg8 puncta was significantly reduced upon TgAtg3 depletion in the iTgAtg3 (non-treatment versus treatment: 91.5 ± 1.72% versus 17.8 ± 1.48%, *P* < 0.0001) and 3HA-TgAtg3^Mut^c (88.2 ± 1.16% versus 14.1 ± 0.70%, *P* < 0.0001) lines compared with the parental (91.5 ± 1.72% versus 85.0 ± 4.16%, *P* = 0.8754) and 3HA-TgAtg3^WT^c (93.5 ± 1.57% versus 77.6 ± 8.20%, *P* = 0.0776) lines (Fig. 2C and D). Taken together, these results suggested that mutated TgAtg3^F239A/I242A^ can impair TgAtg8-conjugation capacity in the autophagosomes.

### TgAtg3 AIM mutants show reduced plaque formation and delayed death phenotype.

**FIG 3:**
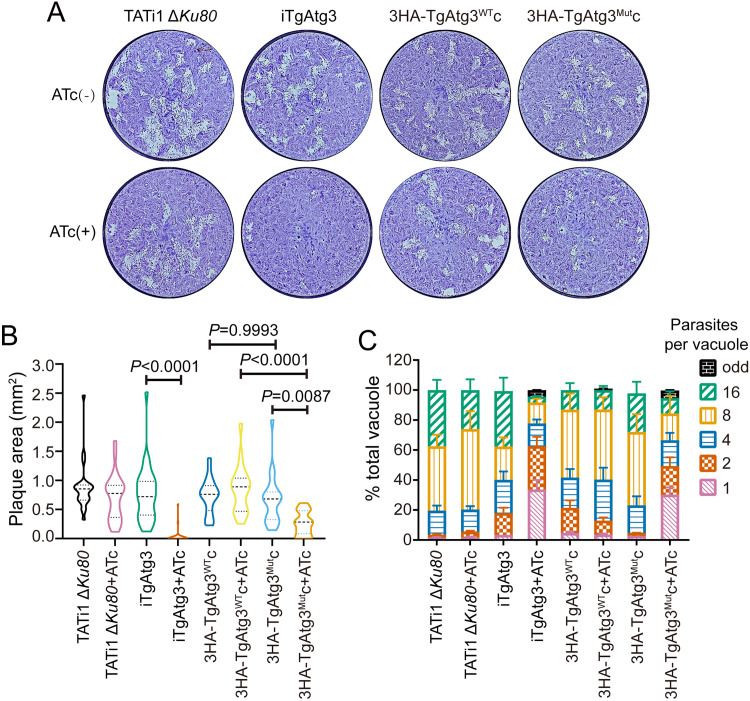
Parasites expressing TgAtg3 AIM mutation show a pronounced growth defect. (A) Plaque assays were performed by infecting HFFs with each line for 7 days, with or without ATc. (B) Measurements of lysis plaque areas via ImageJ show a significant defect in both iTgAtg3 and 3HA-TgAtg3^Mut^c lines compared to the parental and 3HA-TgAtg3^Mut^c lines. Data are means ± SEM from three biological replicates. Statistical analysis was done using one-way ANOVA. (C) Parasites treated with ATc for 48 h were allowed to reinvade new HFFs in the presence of ATc. Parasite replication was assessed at 24 h by counting the number of parasites per vacuole, each with at least 200 parasites per line counted. Data are means ± SEM from three biological replicates. Statistical analysis was done using one-way ANOVA.

To investigate the impact of TgAtg8-TgAtg3 interaction on parasite proliferation, we compared plaque sizes by culturing these cell lines in the absence or presence of ATc for 7 days. Plaque sizes in the iTgAtg3 (non-ATc treatment versus ATc treatment: 0.76 ± 0.08 versus 0.03 ± 0.02) and 3HA-TgAtg3^Mut^c (0.67 ± 0.09 versus 0.26 ± 0.04) parasites, but not in the parental (0.88 ± 0.09 versus 0.70 ± 0.08) or 3HA-TgAtg3^WT^c (0.73 ± 0.07 versus 0.85 ± 0.10) parasites, were severely impaired in the presence of ATc (Fig. 3A and B). Since previous work revealed that TgAtg3-deficient parasites show a delayed death phenotype but have no detectable defects in invasion and egress (14), we performed an intracellular growth assay to assess the rate of parasite division between lines more precisely. The delayed death phenotype means a that significant defect in parasite replication is detected only after parasite egress from the first host cell and invasion of the second one. Thus, all lines were preincubated with ATc for 48 h and then allowed to invade human foreskin fibroblasts (HFFs). Cultures were fixed 24 h after ATc treatment and the numbers of parasites per vacuole were counted. We statistically analyzed the proportions of all vacuoles containing 1 to 4 parasites in each group and observed a significant growth defect in the iTgAtg3 (non-ATc treatment versus ATc treatment: 0.39 ± 0.04 versus 0.79 ± 0.04, *P* = 0.0002) and 3HA-TgAtg3^Mut^c (0.21 ± 0.04 versus 0.63 ± 0.04, *P* = 0.0001) parasites upon ATc treatment compared with the parental (0.20 ± 0.05 versus 0.21 ± 0.04, *P* > 0.9999) and 3HA-TgAtg3^WT^c parasites (0.41 ± 0.03 versus 0.41 ± 0.05, *P* > 0.9999) (Fig. 3C). In addition, compared with parental parasites, we found that there was no statistical difference in iTAtg3 (*P* = 0.114), 3HA-TgAtg3^WT^c (*P* = 0.059) and 3HA-TgAtg3^Mut^c (*P* > 0.9999) without ATc treatment, suggesting that their defect in parasite replication resulted from the knockdown of TgAtg3 rather than from genetic modifications. Taken together, these results validated the key role of the AIM of TgAtg3 in parasite intracellular replication.

### TgAtg3 AIM mutants display a significant defect in apicoplast homeostasis rather than in mitochondrial network.

**FIG 4:**
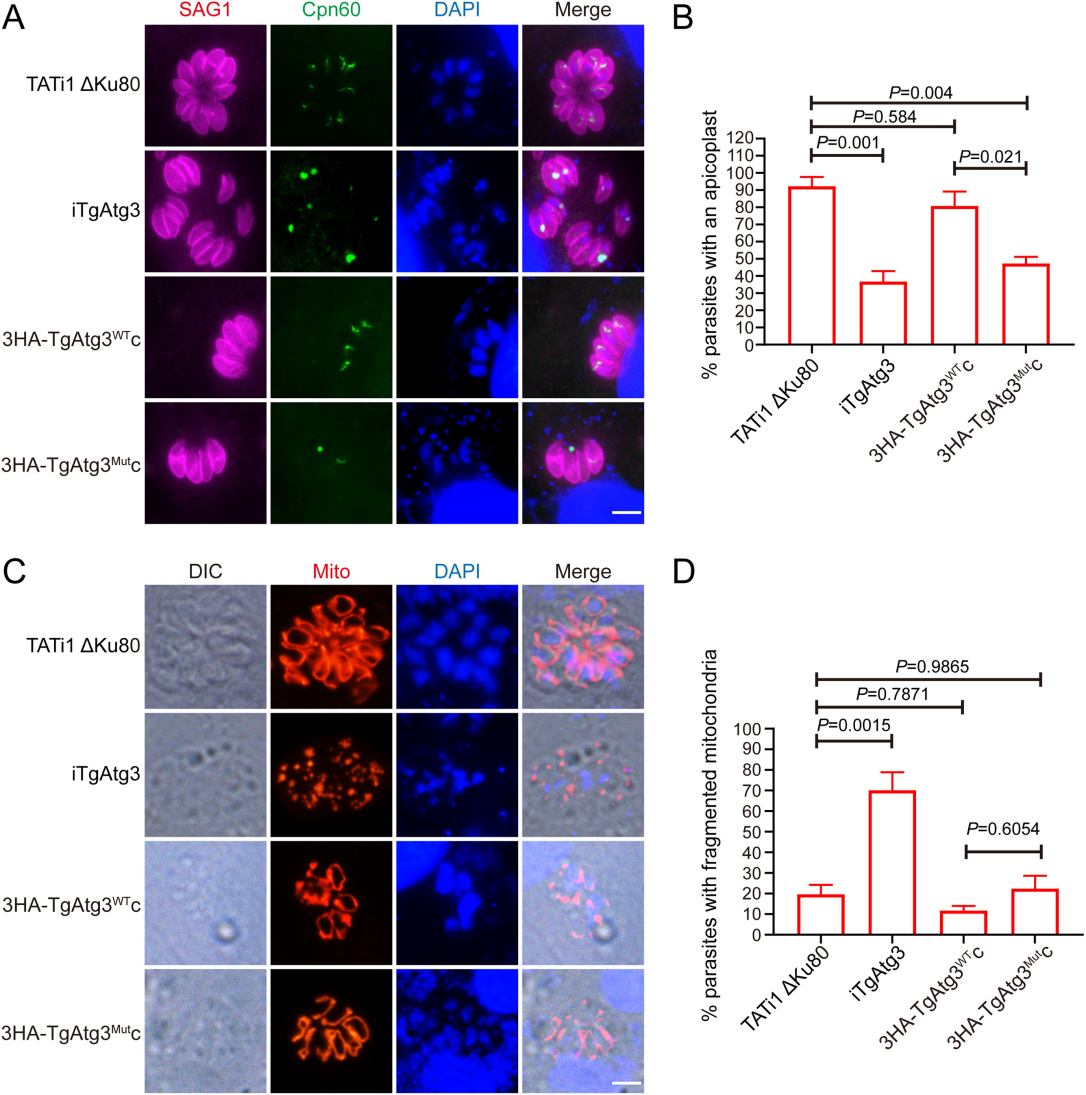
Parasites expressing TgAtg3 AIM mutation show a defect in apicoplast inheritance rather than in mitochondrial integrity. (A) IFA assay to detect the apicoplast marker TgCpn60 in HFFs infected with each line treated with ATc for 48 h. Scale bar = 5 μm. (B) Quantification of percentages of tachyzoites with an apicoplast after incubation with ATc for 48 h. Data are means ± SEM from three biological replicates, each with at least 200 parasites per line counted. Statistical analysis was done using one-way ANOVA. (C) Analysis of iTgAtg3 and complemented cell lines, transiently expressing TgHsp60-RFP, which were preincubated for 48 h with ATc and made to invade new HFFs for 24 h before being fixed and processed for IFA. Scale bar = 5 μm. (D) Quantification of percentages of tachyzoites with fragmented mitochondria after incubation with ATc for 48 h. Data are means ± SEM from three biological replicates, each with at least 200 parasites per line counted. Statistical analysis was done using one-way ANOVA.

Since parasite growth was severely dampened in the 3HA-TgAtg3^Mut^c line following endogenous TgAtg3 knockdown, we sought to investigate whether this was associated with the apicoplast inheritance defect by performing IFA using an antibody against TgCpn60, an apicoplast matrix protein marker. No statistical differences were observed in the absence of ATc (Fig. S2), suggesting that genetic modification did not impair apicoplast inheritance. In contrast, we noticed that compared to parental parasites (92.2 ± 5.49%), more than 60% of the iTgAtg3 (36.7 ± 6.12%) parasites lost their apicoplast and an accumulation of apicoplasts within residual bodies after ATc treatment, which is consistent with previous findings (23). Again, this defect was partially restored in the 3HA-TgAtg3^WT^c (80.8 ± 8.37%) lines but not in the 3HA-TgAtg3^Mut^c lines (47.3 ± 3.81%) (Fig. 4A and B).

Given that TgAtg3 has also proven to maintain mitochondrial homeostasis (14), we further evaluated mitochondrial morphology by IFA assay. Individual cell lines were treated for 48 h with ATc and then transiently transfected with a plasmid mito-RFP, in which red flourescent protein (RFP) was fused to the mitochondrial matrix-targeting leader sequence of TgHsp60 (31). As expected, we observed that a significant number of parasites appeared to have fragmented mitochondrion in the iTgAtg3 (70.1 ± 8.75%) line. However, no obvious defect was discoverable in either the parental (19.6 ± 4.61%), 3HA-TgAtg3^WT^c (11.7 ± 2.30%), or 3HA-TgAtg3^Mut^c lines (22.4 ± 6.26%) (Fig. 4C and D). Therefore, we assume that disruption of the TgAtg8-TgAtg3 interaction specifically impairs apicoplast inheritance rather than mitochondrial maintenance.

### Disruption of TgAtg8-TgAtg3 interaction hinders TgAtg8 apicoplast localization.

**FIG 5:**
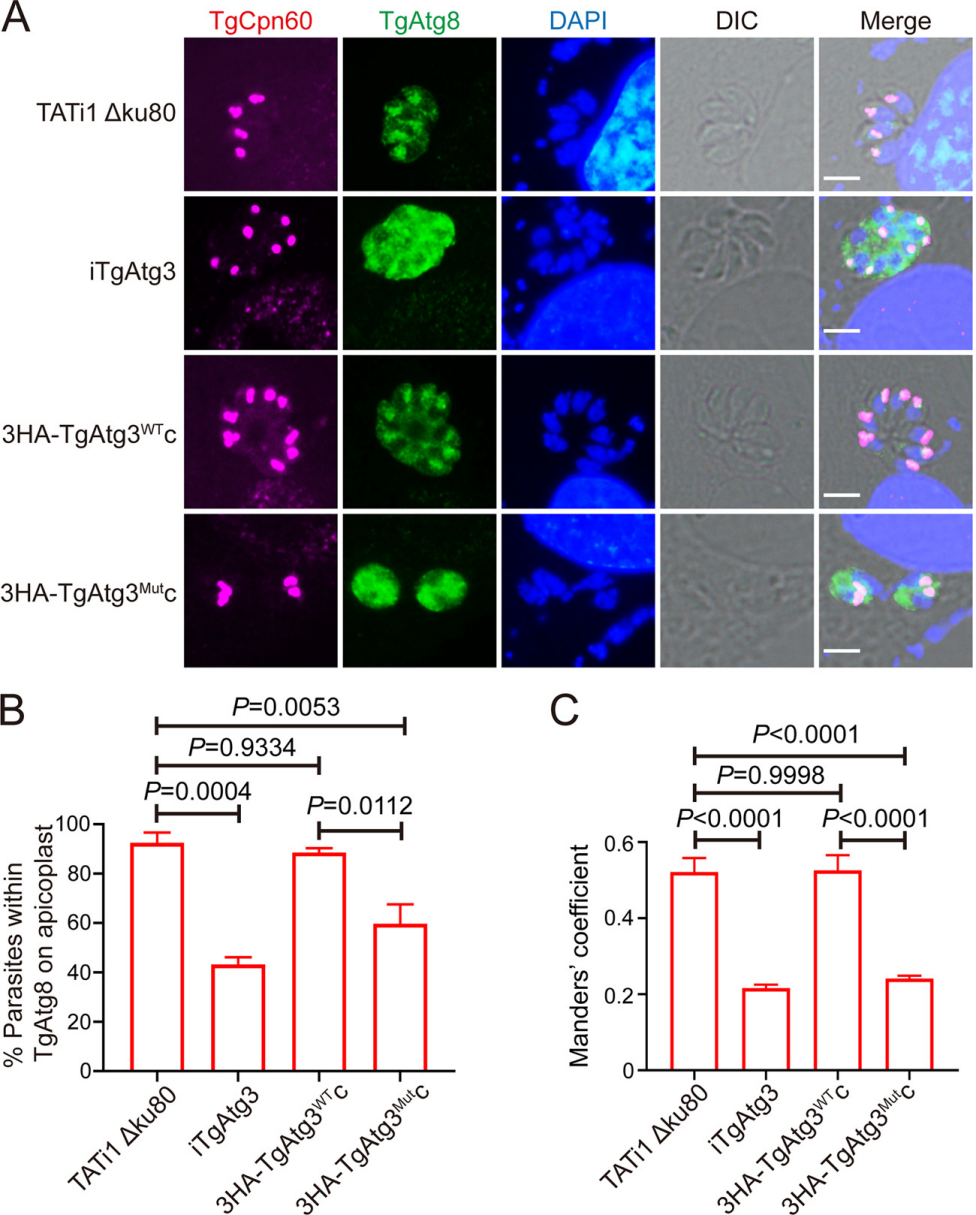
TgAtg8-TgAtg3 interaction regulates TgAtg8 apicoplast localization. (A) IFA was performed to observe TgAtg8 localization after treatment with ATc for 48 h. Scale bar = 5 μm. (B) Number of apicoplast-bearing parasites that displayed the presence of TgAtg8 on apicoplast. Data are means ± SEM from two biological replicates. Statistical analysis was done using one-way ANOVA. (C) Cytosolic localization of TgAtg8 was quantified using the thresholded Manders’ coefficients (TgAtg8 overlapping with the apicoplast) (*n* = 30). Thresholded Manders’ coefficients were calculated using the ImageJ plugin Color C2. Statistical analysis was done using one-way ANOVA.

To assess the impact of disruption of TgAtg8-TgAtg3 interaction on TgAtg8 targeting to the apicoplast, individual cell lines were treated for 48 h in the absence or presence of ATc. We found that TgAtg8 is able to localize on the apicoplast in all cell lines in the absence of ATc (Fig. S3). After ATc treatment, however, while TgAtg8 colocalized with TgCpn60 in parental (92.5 ± 4.15%) and 3HA-TgAtg3^WT^c parasites (88.5 ± 1.74%), its localization to the apicoplast was hindered in a significant number of iTgAtg3 (43.2 ± 2.98%) and 3HA-TgAtg3^Mut^c (59.7 ± 7.80%) parasites (Fig. 5A and B). Additionally, a concomitant increase of TgAtg8 in parasite cytoplasm was observed, as a significant reduction in Manders’ coefficient (TgAtg8 overlapping with the apicoplast) was shown in both the iTgAtg3 (0.22 ± 0.01) and 3HA-TgAtg3^Mut^c strains (0.24 ± 0.01), but not in the 3HA-TgAtg3^WT^c (0.53 ± 0.04) strain, compared to parental parasites (0.52 ± 0.04) (Fig. 5C). Taken together, these results suggested that TgAtg8-TgAtg3 interaction may facilitate TgAtg8 apicoplast localization, which regulates apicoplast inheritance.

### The mutation of R27E in TgAtg8 inhibited intracellular growth of tachyzoites.

**FIG 6:**
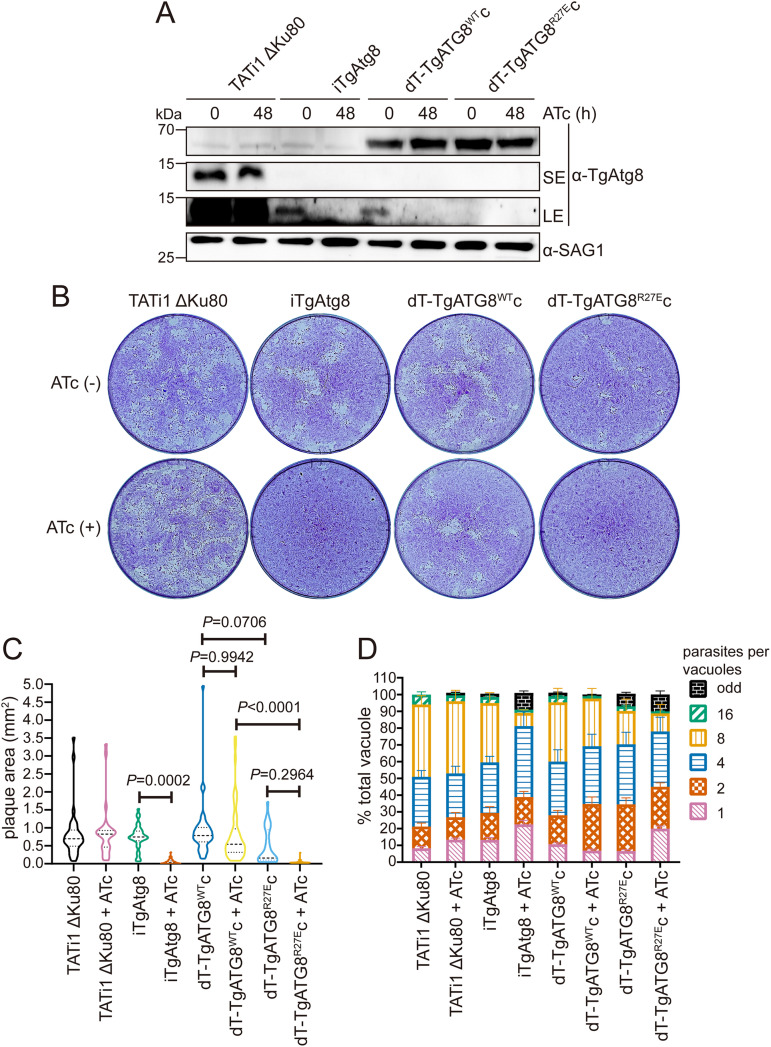
Parasites expressing TgAtg8 R27E mutation show a pronounced growth defect. (A) WB analyses detecting TgAtg8 expression in each line preincubated with or without ATc for 48 h using anti-TgAtg8 antibody. Anti-SAG1 antibody was used as a loading control. SE, short exposure; LE, long exposure. (B) Plaque assays were performed by infecting HFFs with each line for 7 days, with or without ATc. (C) Measurements of lysis plaque areas via ImageJ show a significant defect in both iTgAtg8 and dT-TgAtg8^R27E^c lines compared to the parental and dT-TgAtg8^Mut^c lines. Data are means ± SEM from three biological replicates. Statistical analysis was done using one-way ANOVA. (D) Parasites treated with ATc for 48 h were allowed to reinvade new HFFs in the presence of ATc. Parasites replication was assessed at 24 h by counting parasites per vacuole. Data are means ± SEM from three biological replicates, each with at least 200 vacuoles per line counted. Statistical analysis was done using one-way ANOVA.

We have previously demonstrated that the R27 residue in TgAtg8 plays a key role in mediating TgAtg8-TgAtg3 interaction *in vitro* using biochemical experiments based on site-directed mutation (28). To test whether this residue influences TgAtg8 lipidation, we generated an inducible knockdown line of TgAtg8 (iTgAtg8) by replacing the native promoter with an Atc-regulated promoter, as described previously (18). A pyrimethamine-resistant clone was validated for correct genomic integration by diagnostic PCR (Fig. S4A). We next complemented the iTgAtg8 line by integrating into the genome an additional wild-type gene or mutant allele which harbored an R27E point mutation, fused with a tandem dimer of Tomato (dT) at the 5′-end of the *TgAtg8* gene made to express, driven by *tubulin* promoter. The two complemented cell lines were named dT-TgATG8^WT^c and dT-TgATG8^R27E^c, respectively. RT-qPCR analyses showed that *TgAtg8* transcription in the iTgAtg8 line was significantly downregulated 48 h after ATc treatment, while both complemented lines displayed *TgAtg8* transcriptional levels similar to that of the parental line even after addition of ATc (Fig. S4B). To examine the extent of TgAtg8 knockdown upon the addition of ATc, we cultured all cell lines in the absence or presence of ATc for 48 h, then separated proteins by WB assays. Protein levels of endogenous TgAtg8 in iTgAtg8 were significantly depleted after ATc treatment, while both complemented lines exhibit an expected band at the molecular mass of 62 kDa, irrespective of ATc treatment (Fig. 6A). In accordance with the previous description (18), however, we also found that the endogenous TgAtg8 was very weakly expressed in iTgAtg8 and the two complemented lines, even without ATc treatment (Fig. 6A), presumably due to the replacement of the native *TgAtg8* promoter with the weaker inducible *Sag4* promoter. In spite of the weak expression of endogenous TgAtg8, the previous study demonstrated that a low level of TgAtg8 is adequate to achieve its function and that its downregulation is efficiently induced by addition of ATc (18). We thus decided to utilize the three transgenic lines for follow-up experiments.

To investigate the impact of R27 of TgAtg8 on parasite proliferation, we grew all cell lines in the absence or presence of ATc for 7 days and compared plaque sizes. Plaque sizes in iTgAtg8 (non-treatment versus treatment: 0.75 ± 0.06 versus 0.05 ± 0.01) parasites, but not in parental (0.83 ± 0.12 versus 0.88 ± 0.14) parasites, were severely impaired in the presence of ATc. Complementation with constitutively expressed dT-TgAtg8^WT^ (non-treatment versus treatment: 0.92 ± 0.12 versus 0.82 ± 0.11) but not with dT-TgAtg8^R27E^ (0.47 ± 0.10 versus 0.05 ± 0.01) restored plaque formation in iTgAtg8 parasites cultured in the presence of ATc (Fig. 6B and C), indicating that (i) the proliferation defect we observed upon endogenous TgAtg8 knockdown was specifically due to loss of TgAtg8 and (ii) R27 residue plays an important role in regulation of function of TgAtg8. However, we noticed that the dT-TgATG8^R27E^c cell lines showed a noticeable defect in plaque formation compared to dT-TgATG8^WT^c, even without ATc treatment, suggesting that constitutively expressed TgAtg8 mutant may competitively inhibit endogenous TgAtg8 function. Given that TgAtg8 depletion leads to defects in the intracellular division of parasites (18), we further assessed the function of R27 of TgAtg8 by intracellular growth assays. All cell lines, preincubated with ATc for 48 h, were allowed to invade HFFs in the presence of ATc for another 24 h. We found that in the next lytic cycle (72 h after ATc treatment), almost 80% of the vacuoles in ATc-treated iTgAtg8 (non-treatment versus treatment: 39.2 ± 3.27 versus 76.6 ± 2.22, *P* < 0.0001) contained one, two, or four parasites, while untreated parasites continued to replicate as well as the parental (18.94 ± 1.40 versus 20.8 ± 1.33, *P* = 0.9999) parasites did (Fig. 6D). As with the results of plaque assay, the division defects could be rescued by complementing dT-TgAtg8^WT^ (non-ATc treatment versus ATc treatment: 42.1 ± 2.64 versus 39.94 ± 5.01, *P* = 0.9996), but not dT-TgAtg8^R27E^ (22.3 ± 3.82 versus 65.6 ± 4.03, *P* < 0.0001), highlighting the importance of R27 in TgAtg8.

### The mutation of R27E in TgAtg8 disrupts the apicoplast inheritance.

**FIG 7:**
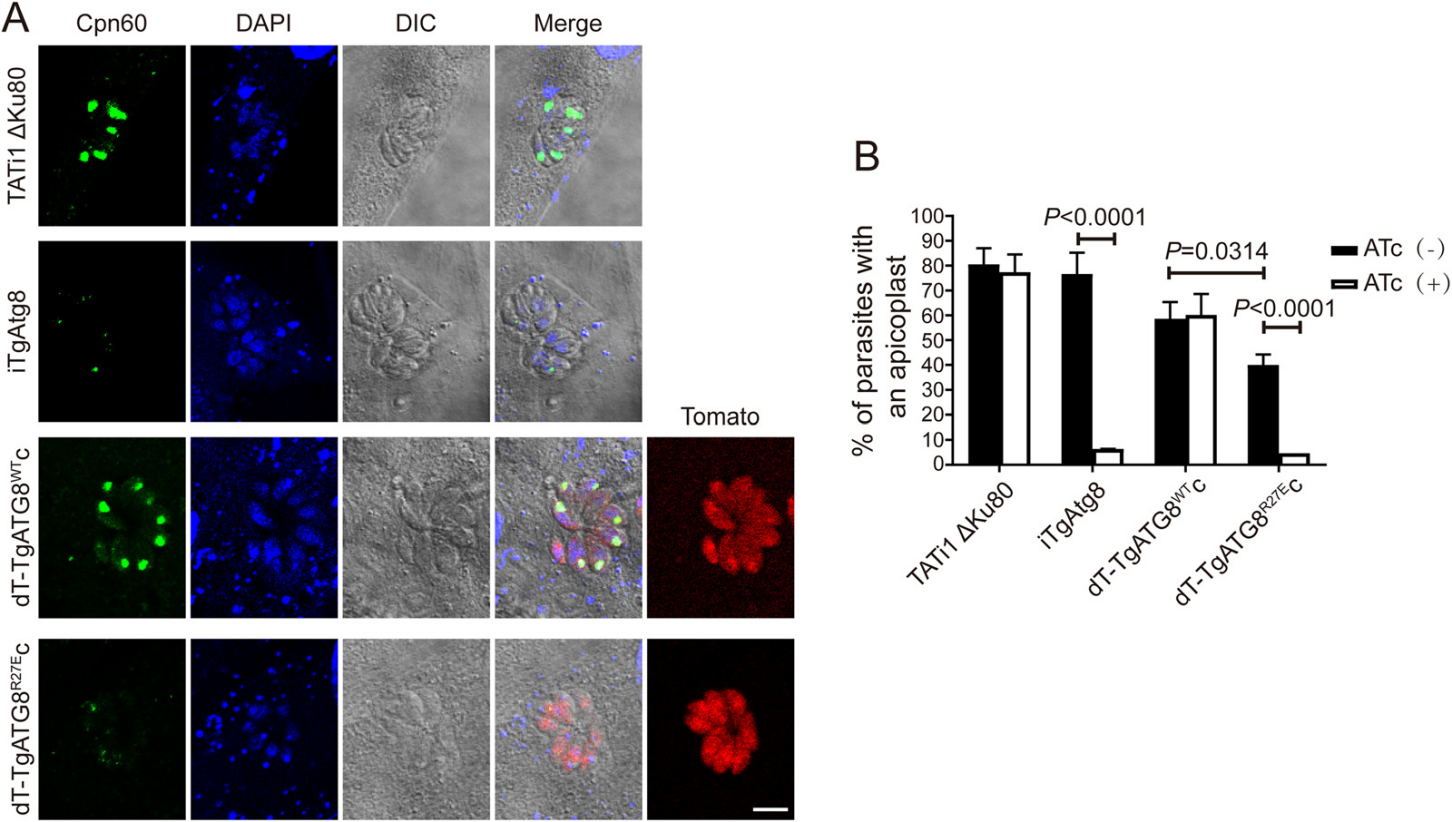
Parasites expressing TgAtg8 R27E mutation show a defect in apicoplast inheritance. (A) IFA assay to detect the apicoplast marker TgCpn60 in HFFs infected with each line treated with ATc for 48 h. Scale bar = 5 μm. (B) Quantification of percentages of tachyzoites with an apicoplast after incubation with or without ATc for 48 h. Data are means ± SEM from three biological replicates, each with at least 200 parasites per line counted. Statistical analysis was done using one-way ANOVA.

Since the function of TgAtg8 has been associated with apicoplast inheritance (18) and R27 in TgAtg8 has been suggested to be involved in mediating TgAtg8-TgAtg3 interaction (28), the role of R27 in TgAtg8 in apicoplast inheritance was investigated by staining parasites with anti-Cpn60. As expected, in the iTgAtg8 line treated with ATc for 96 h, TgAtg8 depletion resulted in apicoplast loss in a significant number of parasites compared with parasites in the absence of ATc (6.4 ± 0.06% versus 76.6 ± 8.6%, *P* < 0.0001). For both complemented lines, a constitutively expressed copy with wild-type TgAtg8 partially rescued this phenotype upon ATc treatment, and no statistical differences were observed between the ATc-treated and untreated groups (60.2 ± 8.4% versus 58.6 ± 6.8%, *P* > 0.05); whereas the R27 mutant did not rescue this phenotype, because the percentage of parasites containing apicoplast was remarkably reduced in the ATc-treated group compared with the untreated group (4.7 ± 0.03% versus 40.0 ± 4.2%, *P* < 0.0001) (Fig. 7A and B). Likewise, we noticed that the apicoplast was significantly lost in the dT-TgAtg8^R27E^ complemented cell lines compared with the dT-TgAtg8^WT^ complemented cell lines, even without ATc treatment (*P* < 0.05). Therefore, combining the results from plaque assays and intracellular assays, it is likely that constitutively expressed TgAtg8^R27E^ impairs the proliferation ability of parasites by competitively inhibiting endogenous TgAtg8 function in apicoplast inheritance. Overall, the rapid and specific loss of the apicoplast in dT-TgAtg8^R27E^ complemented cell lines suggests an important role of TgAtg8-TgAtg3 interaction in the regulation of apicoplast inheritance.

## DISCUSSION

Except for in *Toxoplasma* and *Plasmodium*, there is no direct evidence so far to confirm whether the Atg8-Atg3 interaction is attributed to parasite growth and replication. In Plasmodium falciparum, even though numerous small molecule inhibitors identified for blocking the recombinant PfAtg8-PfAtg3 interaction through *in vitro* high-throughput screens could inhibit parasite growth in both blood- and liver-stage cultures (25, 26), the possibility that these compounds inhibited parasite growth via indirect off-target effects was not excluded. In *Toxoplasma*, a previous study produced an iTgAtg3 line based on the Tet-off system and demonstrated that TgAtg3 depletion impairs TgAtg8 lipidation (14) and apicoplast inheritance (23). However, it is difficult to differentiate whether this phenotype resulted from specifically hampering the TgAtg8-TgAtg3 interaction or from disruption of the noncanonical function of TgAtg3, since TgAtg3 depletion also causes a dramatic collapse of the parasite mitochondrion (14). Additionally, although we previously confirmed that blockage of the TgAtg8-TgAtg3 interaction significantly weakens TgAtg8 lipidation using mutagenesis and biochemical analyses *in vitro* (28), the functions of this interaction in regulating parasite’s growth are not illustrated. Therefore, it is needed to investigate whether TgAtg8-TgAtg3 interaction plays an important role in apicoplast inheritance and parasite growth.

In this study, we disrupted TgAtg8-TgAtg3 interaction in the tachyzoite using a mutagenesis strategy. Our results validate previous findings that the core AIM of TgAtg3, ^239^FADI^242^, is responsible for mediating TgAtg8-TgAtg3 interaction, and show that blocking this interaction induces the loss of apicoplast and the delayed death phenotype in tachyzoites rather than mitochondrial fragmentation.

Within an intermediate host, Toxoplasma gondii can differentiate from a tachyzoite form to a tissue cystic structure (containing the bradyzoite form) and vice versa (32). Tachyzoites and bradyzoites are responsible for the acute and chronic forms of toxoplasmosis, respectively. Several medicines are currently available for clinical treatment of acute toxoplasmosis, but they are ineffective against cysts and fail to prevent the reactivation of latent toxoplasmosis (33). Therefore, it is desirable to identify an effective therapeutic target against both tachyzoites and bradyzoites. In tachyzoites, the essentiality of TgAtg8 seems to be solely attributed to parasite apicoplast function (18, 34). The reasonable explanation is that parasites can acquire sufficient nutrients from the host cell cytoplasm across a parasitophorous vacuole (PV) membrane throughout the tachyzoite phase. Thus, the canonical autophagy pathway might be dispensable for tachyzoite growth and replication within the PV. This hypothesis has been supported by disrupting TgAtg9 (35) and TgCPL (36, 37), respectively. Atg9 is a key component of the Atg2/18/9 complex that facilitates the development of the autophagosome precursor and is regarded as a major landmark of canonical autophagy pathway (38–40). However, in the *Toxoplasma* tachyzoite, it has been determined that TgAtg9 was nonessential in cultured parasites and that TgAtg9-deficient parasites displayed a non-defect in apicoplast inheritance (35). TgCPL is a cathepsin l-enzyme responsible for the degradation of exogenous and endogenous proteins in the plant-like vacuole/vacuolar compartment (VAC), a lysosome-like organelle (17). Similar to TgAtg9 deficiency, the ablation of TgCPL had no observable impact on apicoplast inheritance and tachyzoite growth (36). Likewise, in *Plasmodium*, although PfAtg8 has been proposed to be involved in diverse processes, such as starvation-induced autophagy, stage-specific organelle turnover, and intracellular vesicle trafficking (41–43), it has been verified that the only essentiality of PfAtg8 is attributed to the parasite’s apicoplast biogenesis (44). These findings indicate that apicomplexan Atg8 homologs seem to be clearly different from yeast and mammalian Atg8s, which are not strictly necessary for cell growth under nutrient-rich conditions (45, 46). However, a recent work has shown that, in *Toxoplasma* bradyzoites,TgAtg8 also benefits bradyzoite viability due to its abilities of efficient autophagosome formation and transportation of autophagic materials to VAC (16). Therefore, the dual functions of TgAtg8, apicoplast biogenesis in tachyzoites and viability in bradyzoites, suggest that it may be leveraged for the development of drugs against both acute and chronic *Toxoplasma* infection.

In *Plasmodium* and *Toxoplasma*, there is clear evidence that localization of Atg8 to either the outer membrane of the apicoplast or to autophagosome-like structures depends upon its conjugation to PE, highlighting a vital function of Atg8 conjugation machinery in apicomplexan parasite biology. In this machinery, the Atg8-Atg3 interaction is an important modulator of the conjugation of Atg8 to PE and has been applied as novel target for the screening of antimalarial drugs (27). These findings demonstrate the possibility of specifically inhibiting the Atg8-Atg3 interaction in apicomplexan parasites and suggest that the interaction may be an attractive target both for therapeutics and for validation of Atg8 function in apicomplexan-specific biological processes.

Our results also suggest that the TgAtg8-TgAtg3 interaction may be dispensable for the maintainance of mitochondrial integrity. However, many questions remain unanswered regarding the function of the machinery regulating TgAtg8 membrane association. For instance, depletion of either TgAtg3 or TgAtg4 leads to a significantly fragmented mitochondrion in tachyzoites. In contrast, when depleted of TgAtg8, tachyzoites did not display a significant alteration of the mitochondrial network compared with that of wild-type parasites. Since the apicoplast and mitochondrion are spatially close in the parasite, the depolarized mitochondrion observed in TgAtg3 and TgAtg4 mutants might be due to a consequence of apicoplast loss rather than a direct effect. Another possible explanation is that mutation of either AIM of TgAtg3 or R27 of TgAtg8 can only weaken, not eliminate, the TgAtg8-TgAtg3 interaction. Also, it needs to be investigated whether TgAtg3 and TgAtg4 possess other functional regions regulating the mitochondrial network. Again, *in vivo* studies using both the TgAtg3^Mut^ and TgAtg8^R27E^ complemented lines we have generated in this study could reveal more interesting features.

In summary, our study has provided firm evidence for the occurrence of a functional TgAtg8-TgAtg3 interaction in the apicomplexan parasite Toxoplasma gondii. Its function is crucial for maintaining apicoplast inheritance and parasite growth. More work is needed to definitively demonstrate that there is a fully functional TgAtg8-TgAtg3 interaction in *Toxoplasma* bradyzoite viability. However, considering our data and the significant function of TgAtg8 in bradyzoite viability, the TgAtg8-TgAtg3 interaction could be considered a possible parasitic drug target against both tachyzoites and bradyzoites of Toxoplasma gondii, especially given the apparent peculiarities in the regulatory mechanism of TgAtg8-TgAtg3 interaction compared with its host counterpart.

## MATERIALS AND METHODS

### Primers and antibodies.

All primers and antibodies used in this study are listed in the supplemental material in Table S1 and Table S2, respectively.

### Host cells and parasite culture.

HFFs (ATCC CRL-1634) were grown in Dulbecco’s modified Eagle’s medium (Gibco-BRL) supplemented with 10% fetal bovine serum (Gibco), 100 U/mL penicillin, and 100 mg/mL streptomycin. Cells were maintained in a 37°C humidified incubator with 5% CO_2_. Tachyzoites of the TATi1 Δ*Ku80* strain (29) and derived transgenic parasites were maintained by serial passage in HFF monolayers.

### Generation of conditional knockdown and complemented cell lines.

Both iTgAtg3 and iTgAtg8 cell lines were generated based on the Tet-off system.

To obtain the iTgAtg3 cell line, a 1416-bp fragment corresponding to the 5′-end of *TgAtg3* (TGGT1_236110), starting with the initiation codon, was amplified from genomic DNA by PCR using KAPA HiFi HotStart DNA polymerase (Kapa Biosystems, Inc., USA) with the primers P1/P2. The fragment was then cloned into BglII/NotI site of the pDHFR-TetO7Sag4-TgAtg8 (18) to replace the 5′-region of *TgAtg8* (TGGT1_254120). The TATi1 Δ*Ku80* was transfected with 100 μg of the XcmI-linearized pDHFR-TetO7Sag4-TgAtg3. After 24 h, transgenic parasites were selected with 10 μM pyrimethamine and cloned by limiting dilution. Clones were confirmed by either PCR1 (P3/P4) or PCR2 (P3/P5) and PCR3 (P4/P6) to detect the presence of endogenous or recombined locus. To generate the complemented cell lines using CRISPR/Cas9, the coding sequences of TgAtg3^WT^ and TgAtg3^Mut^ were amplified with the primer pair P7 (containing triple HA-tag)/P8 from the pHis6-TgAtg3 and pHis6-TgAtg3^F239A/I242A^ plasmids (28), respectively, and were cloned into pGFP-TgAtg8 vector (14) with NsiI and PacI to replace GFP-TgAtg8. Meanwhile, the 5′ and 3′ homologous flanks of *UPRT* (TGGT1_312480) were amplified from genomic DNA using the primer pairs P9/P10 and P11/P12, respectively. The two fragments were cloned upstream and downstream of the 3HA-TgAtg3 sequence with ApaI/NsiI and PacI/NotI, respectively. Finally, the two fragments, UPRT5′-3HA-TgAtg3^WT^-UPRT3′ and UPRT5′-3HA-TgAtg3^Mut^-UPRT3′, were amplified separately from the two constructed plasmids above using the primer pair P13/P14, and cotransfected with pSAG1-CAS9-U6sgUPRT plasmid (47) into the iTgAtg3 line. Transgenic parasites were selected using 10 μM fluorodeoxyribose and positive clones were further analyzed by PCR using the primer pair P15/P16 for correct integration at the *UPRT* locus, and by WB for 3HA-TgAtg3 expression.

The iTgAtg8 cell line was generated using the plasmid pDHFR-TetO7Sag4-TgAtg8 as described previously (18). Correct integration in positive clones was confirmed by PCR with the primer pairs P19/P20 and P17/P18, and the presence of endogenous locus was detected with the primer pairs P19/P18. To generate the TgAtg8^WT^ complemented cell line, the iTgAtg8 cell line was transfected with a circular pTub-dT-TgAtg8-CAT plasmid (18) and selected with chloramphenicol. To generate the TgAtg8^R27E^ complemented cell line, site-directed mutagenesis with a Q5 Site-Directed Mutagenesis MOIkit (New England Biolabs) was used with the primer pair P21/P22 on the pTub-dT-TgAtg8-CAT plasmid to modify the sequence corresponding to the ‘R27’ residue (arginine replaced by a glutamic acid). The resultant pTub-dT-TgAtg8^R27E^-CAT plasmid was transfected into the iTgAtg8 line followed by chloramphenicol selection.

### Real-time quantitative PCR for transcriptional analyses.

Confluent HFF were infected with *Toxoplasma* for 1 h, washed to remove extracellular parasites, then incubated in culture medium containing ATc (1 μg/mL) for 24, 48, or 72 h (h). At each time point, RNA was extracted, and cDNA synthesized using HiScript II Q RT SuperMix (Vazyme, Nanjing, China). qPCR amplification of cDNA (100 ng each) was performed using 2.25 μL of each primer pair (10 μM) and 12.5 μL of SYBR Green Master Mix (Vazyme). Reactions were run on a StepOne Plus Real-Time PCR System (Applied Biosystems). The data of targeted genes were normalized to the parasite β-tubulin housekeeping gene, and 2^–ΔΔCT^ values were calculated. Two different sets of primers for the *TgAtg3* and *TgAtg8* genes were used: P23/P24 and P25/P26, respectively. The primers for the *β-tubulin* gene were P27/P28.

### Induction and monitoring of autophagy.

ATc-treated tachyzoites were extracted from lysed cells, washed twice with HBSS, and kept at 37°C for up to 8 h. Autophagosomes were quantified by fluorescence microscopic observation and counting of TgAtg8 punctate signals. At least 150 tachyzoites were counted in each experimental set. Meanwhile, the presence of lipidated TgAtg8 (TgAtg8-PE) was assessed by WB with anti-TgAtg8 antibody after separation by 6 M urea SDS-PAGE. The ability to form lapidated TgAtg8 was quantified with ImageJ as the intensity ratio of TgAtg8-PE to TgAtg8. Three independent experiments were performed.

### *In vivo* immunoprecipitation.

Extracellular iTgAtg3, 3HA-TgAtg3^WT^c, and 3HA-TgAtg3^Mut^c parasites (1 × 10^8^) were collected and resuspended in 50 mM Tris-HCl (pH 7.5) with 1% Triton X-100, 0.1% SDS, 0.5 mM EDTA, 150 mM NaCl, and cOmplete EDTA-free Protease Inhibitor (Roche). Three cycles of freezing/unfreezing were performed to break the cells, followed by incubation for at least 1 h 4°C on a rotating wheel. Ten μL of lysate was collected as “input” for WB analysis. Centrifugation was performed at 16 000 × *g* for 20 min at 4°C to remove intact parasites. Supernatants were then diluted to half in 50 mM Tris-HCl (pH 7.5) with 150 mM NaCl, 0.5 mM EDTA, and cOmplete EDTA-free Protease Inhibitor (Roche). The diluted supernatant was added to 10 μL of HA nanoab magnetic beads (EveryLab, Shanghai, China) and incubated at 4°C for 4 h on a rotating wheel. Immune complexes were separated with SDS-PAGE followed by WB with anti-HA and anti-TgAtg8 antibodies.

### SDS-PAGE and WB analyses.

SDS-PAGE and WB were performed as described previously (28). SDS-PAGE samples were separated on 15% (vol/vol) and 10% (vol/vol) acrylamide gel for TgAtg8 and TgAtg3, respectively. Primary antibodies used included rabbit anti-TgAtg8 (1:500), rabbit anti-TgAtg3 (1:1,000), mouse anti-TgSAG1 (1:2,000), TgβTubulin (1:1,000), and rabbit anti-HA (1:2,000). The secondary antibodies used were HRP-conjugated goat anti-rabbit IgG and goat anti-mouse IgG, respectively.

### Immunofluorescence assays.

Immunofluorescence assays were performed as described previously (28). Primary antibodies used included rabbit anti-TgAtg8 (1:500), rabbit anti-TgAtg3 (1:200), mouse anti-TgSAG1 (1:2,000), mouse anti-TgCpn60 (1:200), and rabbit anti-HA (1:1,000). The secondary antibodies used were goat anti-mouse Alexa Fluor 488 (1:1,000; YEAST) and goat anti-rabbit Alexa Fluor 546 (1:1,000; YEAST). Images were acquired using an Eclipse Ci-L Fluorescence microscope (Nikon, Japan) equipped with a CFI Plan Fluor optical imaging system, a 100×/1.3-numerical-aperture (NA) Nikon lens, a Digital Sight camera connected to PC via DS-L4 control unit, and NIS-Elements F version 4.60.00 software. Adjustments for brightness and contrast were applied uniformly to the entire image.

### Plaque assays.

Standard plaque assays were performed using confluence HFFs in 24-well plates, infected with 100 tachyzoites treated with or without ATc for 7 days. The HFFs were fixed and stained as described previously (48). The plates were scanned, and the areas of plaques were analyzed using ImageJ. To convert the pixel size of the scanned picture to the actual physical size, we used “Straight” to describe the diameter of each well prior to setting the conversion relationship to “Global” through “Analyze Set Scale.” Subsequently, the plaque areas selected with “Freehand Selections” were measured using “Analyze Measure.”

### Intracellular growth.

Fresh egressed tachyzoites pretreated with ATc for 48 h were collected and used to infect HFFs at a multiplicity of infection (MOI) of 4 (parasite/host cell ratio). HFFs were washed with phosphate-buffered saline (PBS) to remove extracellular parasites at 3 h postinfection and fixed with 4% paraformaldehyde in PBS 24 h after that. IFA was conducted to stain the parasite membrane with rabbit anti-SAG1 and the nuclei with DAPI. Images were acquired by fluorescence microscopy and the number of tachyzoites in each vacuole was calculated. A total of 100 vacuoles were counted for the replication assay.

### Statistical analysis.

All data were plotted and analyzed using Prism 7.0 (GraphPad Software, Inc., San Diego, CA, USA) and are presented as the mean of two or three replicates ± standard error of the mean (SEM). Statistical analysis was performed with a two-tailed Student's *t* test or one-way analysis of variance (ANOVA) with Tukey’s *post hoc* test. A *P* value of <0.05 was considered statistically significant.
