# Supplementary material for: Toxoplasma TgAtg8-TgAtg3 Interaction Primarily Contributes to Apicoplast Inheritance and Parasite Growth in Tachyzoite
Source: Microbiol Spectr. 2022 Feb 23;10(1):e01495-21. doi: 10.1128/spectrum.01495-21 (PMC8865545; doi:10.1128/spectrum.01495-21)
Supplement: SUPPLEMENTAL FILE 1 — Supplemental material. Download SPECTRUM01495-21_Supp_1_seq10.pdf, PDF file, 0.4 MB [file spectrum01495-21_supp_1_seq10.pdf]

## Tables

### Table S1. Primers used in this study

| Primer name | Sequence | product sizes (bp) |
|---|---|---|
| P1 | CGTCTGTCTTCAACCAGATCTATGACGAAGCCTCAAGAGACAC | 1416 |
| P2 | AGCTCCACCGCGGTGGCGGCCGCGCATGCCATTACTACACATCAGA | |
| P3 | TCTCGTTTCGCTCAAAGTGTG | P3/P4 : 1775<br>P3/P5 : 1715<br>P6/P4 : 1744 |
| P4 | CCTTCCTTTGGTGGATCAGTC | |
| P5 | CTGCAAGGCGATTAAGTTGG | |
| P6 | GAAAACATCGTGAGGCTGGTAC | |
| P7 | GACCTGGCCATGGCCATGCATTACCCGTACGACGTCCCGGACTACGCTTAT<br>CCCTATGATGTGCCCGATTATGCGTATCCTTACGATGTTCCAGATGCTGCAG<br>CAATGACGAAGCCTCAAGAGACACC | 1281 |
| P8 | TGAGCACAACGGTGATTAATTAACTACTTCTCGTTTTTTGTACTGCGG | |
| P9 | GGCGAATTGGGTACCGGGCCCGTGCCTAGTATCGAAAGCTGTAG | 971 |
| P10 | GACGTCGTACGGGTAATGCATTTTAGAAGCCCTGTGGACAG | |
| P11 | AAAAACGAGAAGTAGTTAATTAAGTCTCTAGTTTTTTTGACAGACCG | 889 |
| P12 | AGTGACACCGCGGTGGCGGCCGCAGAGAAGTTGTGTGCTAGAGGTTC | |
| P13 | GTGCCTAGTATCGAAAGCTGTAG | 3173 |
| P14 | AGAGAAGTTGTGTGCTAGAGGTTC | |
| P15 | GCGATTCCCGTATTGGTC | 1492 |
| P16 | GTTCCTGCCGATGGTGAG | |
| P17 | CTGCTTTCGTCTGTCTTC | P17/P18 : 1554<br>P19/P18 : 1556<br>P19/P20 : 1565 |
| P18 | CAAATGGCTATGTTCGCC | |
| P19 | CCAAACCAGATATTCGCC | |
| P20 | TACGACTCACTATAGGGC | |
| P21 | GTATCCCAACGAAATTCCGGTCATCTGC | 9681 |
| P22 | TTTGCCCGAATGCGGTGT | |
| P23 | AGTTTCACCTCTGCCCCGA | 124 |
| P24 | CTACGCCTTTCCACTGCCA | |
| P25 | CATCTGCGAAAAAGCTCCCC | 195 |
| P26 | GCCTTCGGTGCCGTATTTTC | |
| P27 | ATGTTCCGTGGTCGCATGT | 104 |
| P28 | TTCATGTTGTTGGGAATCCAC | |

**Table S2. The antibodies used throughout this study are indicated.**

| Antibodies | Dilutions | Sources |
|---|---|---|
| Rabbit anti-TgAtg8 | 1:500 for WB and IFA | Laboratory-prepared |
| Rabbit anti-TgAtg3 | 1:1000 for WB; 1:200 for IFA | Laboratory-prepared |
| Mouse anti-TgβTubulin | 1:1000 for WB | Laboratory-prepared |
| Mouse anti-TgCpn60 | 1:200 for IFA | Laboratory-prepared |
| Mouse anti-TgSAG1 | 1:2000 for WB and IFA | Abcam, ab8313 |
| Rabbit anti-HA | 1:2000 for WB; 1:1000 for IFA | Cell Signalling Technology, 3724S |
| Goat anti-rabbit IgG (H+L), HRP-Labelled | 1:5000 for WB | Biosharp, BL003A |
| Goat anti-mouse IgG (H+L), HRP-Labelled | 1:5000 for WB | Biosharp, BL001A |
| Goat anti-mouse Alexa Fluor 488 | 1:1000 for IFA | YEAST, 33106ES60 |
| Goat anti-rabbit Alexa Fluor 546 | 1:1000 for IFA | YEAST, 33112ES60 |

## Figures

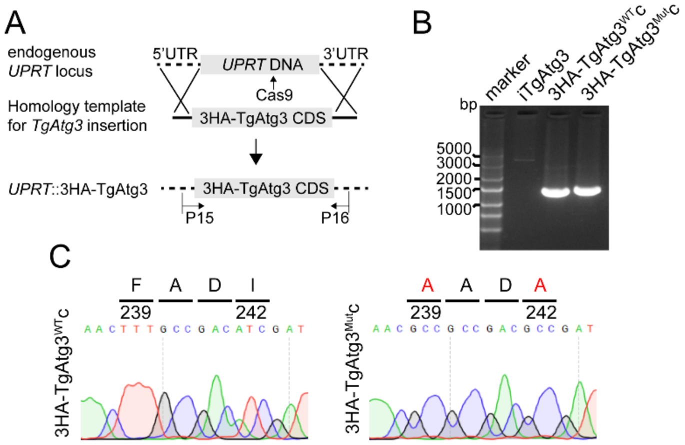


**FIG S1** Validation of two TgAtg3 complemented lines. (A) Schematic representation of generation of the 3HA-TgAtg3$^{WT}$c and 3HA-TgAtg3$^{Mut}$c complemented cell lines by replacement of the endogenous *UPRT* locus. P15 and P16 represent the primer binding sites in the complemented lines (B) Clone derived from the stable FUDR-resistant population was detected for the recombined loci by using diagnostic PCR. iTgAtg3 line is used as control. (C) Sequencing validation of the PCR products from panel A.

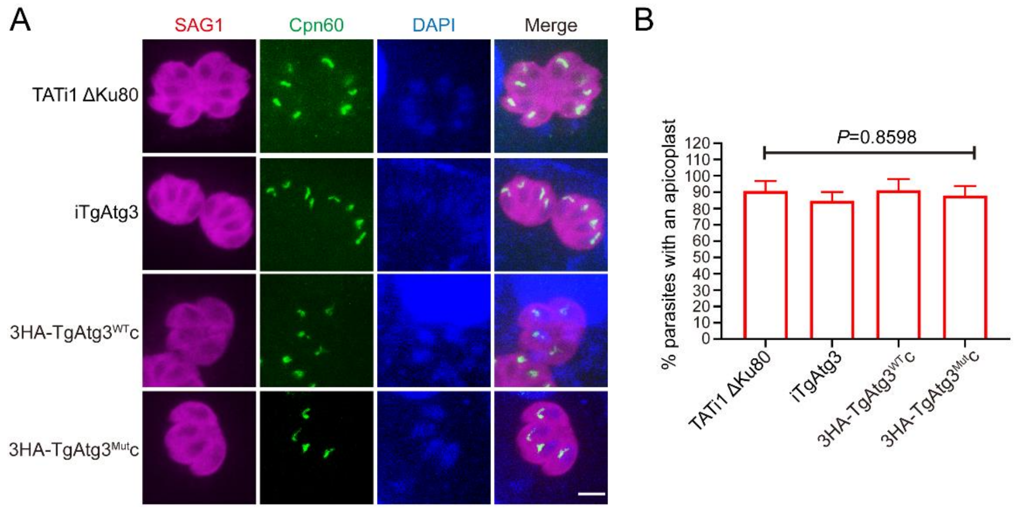


**FIG S2** TgAtg8-TgAtg3 interaction regulates apicoplast inheritance. (A) IFA assay for detection of the apicoplast marker TgCpn60 in HFFs infected with each line in the absence of ATc for 48 h. Scale = 5μm. (B) Quantification of the percentages of tachyzoites with an apicoplast in the absence of ATc for 48 h. Data are means ± SEM from three biologic replicates, each with at least 200 parasites per line counted. Statistical analysis was done using one–way ANOVA.

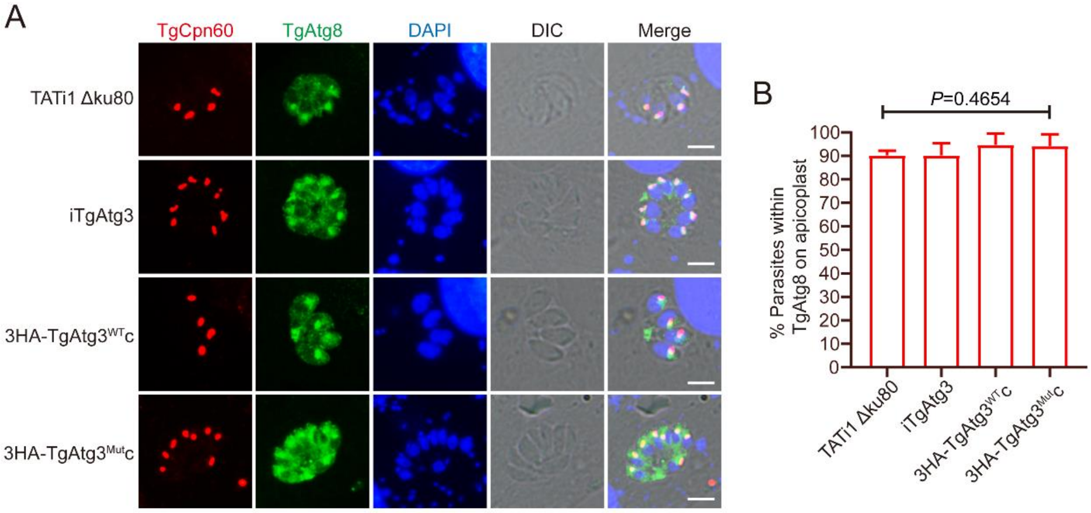


**FIG S3** TgAtg8-TgAtg3 interaction regulates TgAtg8 apicoplast localization. (A) IFA was performed to observe TgAtg8 localization in the absence of ATc for 48 hours. Scale = 5μm. (B) The number of apicoplast-bearing parasites that displayed the presence of TgAtg8 on apicoplast. Data are means ± SEM from two biologic replicates. Statistical analysis was done using one–way ANOVA.

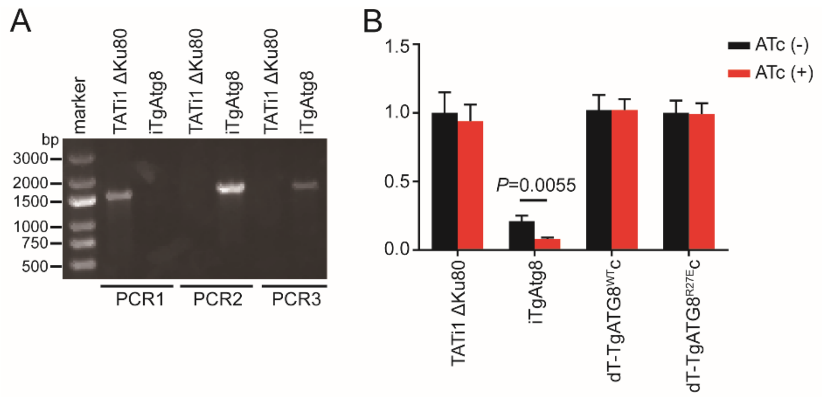


**FIG S4** Generation of iTgAtg8 and complemented lines. (A) Clone derived from a stable pyrimethamine-resistant population was detected for the endogenous and recombined loci by using diagnostic PCR. TATi1 Δ*Ku80* line is used as control. (B) RT-qPCR analyses of *TgAtg8* transcriptional level in each line preceded or not by 48 hours of induction with ATc to regulate expression.
